# Deciphering the Interdomain Coupling in a Gram-Negative Bacterial Membrane Insertase

**DOI:** 10.1021/acs.jpcb.4c02824

**Published:** 2024-09-27

**Authors:** Adithya Polasa, Shadi A. Badiee, Mahmoud Moradi

**Affiliations:** Department of Chemistry and Biochemistry, University of Arkansas, Fayetteville, Arkansas 72701, United States

## Abstract

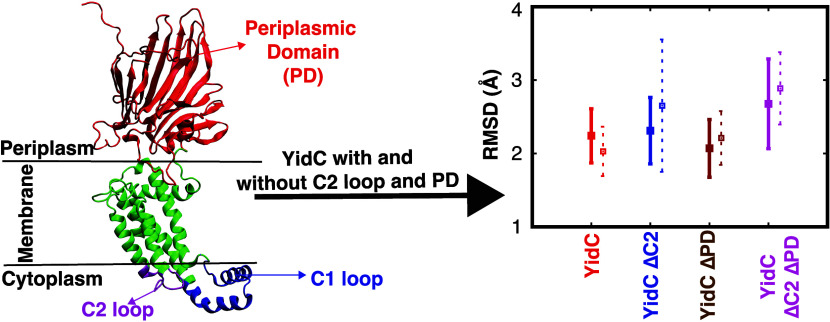

YidC is a membrane protein that plays an important role in inserting newly generated proteins into lipid membranes. The Sec-dependent complex is responsible for inserting proteins into the lipid bilayer in bacteria. YidC facilitates the insertion and folding of membrane proteins, both in conjunction with the Sec complex and independently. Additionally, YidC acts as a chaperone during the folding of proteins. Multiple investigations have conclusively shown that Gram-positive bacterial YidC has Sec-independent insertion mechanisms. Through the use of microsecond-level all-atom molecular dynamics (MD) simulations, we have carried out an in-depth investigation of the YidC protein originating from Gram-negative bacteria. This research sheds light on the significance of multiple domains of the YidC structure at a detailed molecular level by utilizing equilibrium MD simulations. Specifically, multiple models of YidC embedded in the lipid bilayer were constructed to characterize the critical role of the C2 loop and the periplasmic domain (PD) present in Gram-negative YidC, which is absent in its Gram-positive counterpart. Based on our results, the C2 loop plays a role in the overall stabilization of the protein, most notably in the transmembrane (TM) region, and it also has an allosteric influence on the PD region. We have found critical inter- and intradomain interactions that contribute to the stability of the protein and its function. Finally, our study provides a hypothetical Sec-independent insertion mechanism for Gram-negative bacterial YidC.

## Introduction

Membrane proteins participate in crucial biological processes such as signaling, transcriptional regulation, ion transport, proteolysis, motility, metabolism, energy creation, and energy transfer.^1^ Certain specialized cellular machineries in bacteria enable proper membrane protein folding and insertion into the lipid bilayer.^2−4^ Many membrane proteins are inserted through the Sec apparatus. YidC is one of the proteins working with Sec machinery in order to introduce client proteins into the membrane.^2−8^

YidC is a member of the Oxa/Alb3/YidC family of insertases found in mitochondria, chloroplasts, and bacteria.^9−15^ YidC facilitates the cotranslational insertion of membrane proteins into the lipid bilayer. This process occurs as the proteins are being synthesized, ensuring their proper integration into the membrane.^16^ It also plays a vital role in inserting and positioning membrane proteins in bacteria.^17−19^ Insertase proteins, such as YidC, have been exhaustively investigated to determine their importance for inserting proteins into membranes.^20^ In addition to YidC, other bacterial insertase proteins include the SecYEG complex and the Omp85 (also known as the BamA) complex. These insertase proteins play crucial roles in the insertion and folding of membrane proteins into the lipid bilayer. Many researchers have found evidence of YidC in conjunction with the Sec complex,^2−8,21^ which acts to insert peptides into the membrane bilayer through the signal recognition particle (SRP) mechanism. In addition, YidC can fold and insert polypeptides without relying on the Sec-dependent pathway.^3,22−29^ It is particularly essential for the insertion of small phage coat proteins, such as Pf3 coat and M13, via a Sec-independent mechanism.^22,30−35^

**Figure 1:**
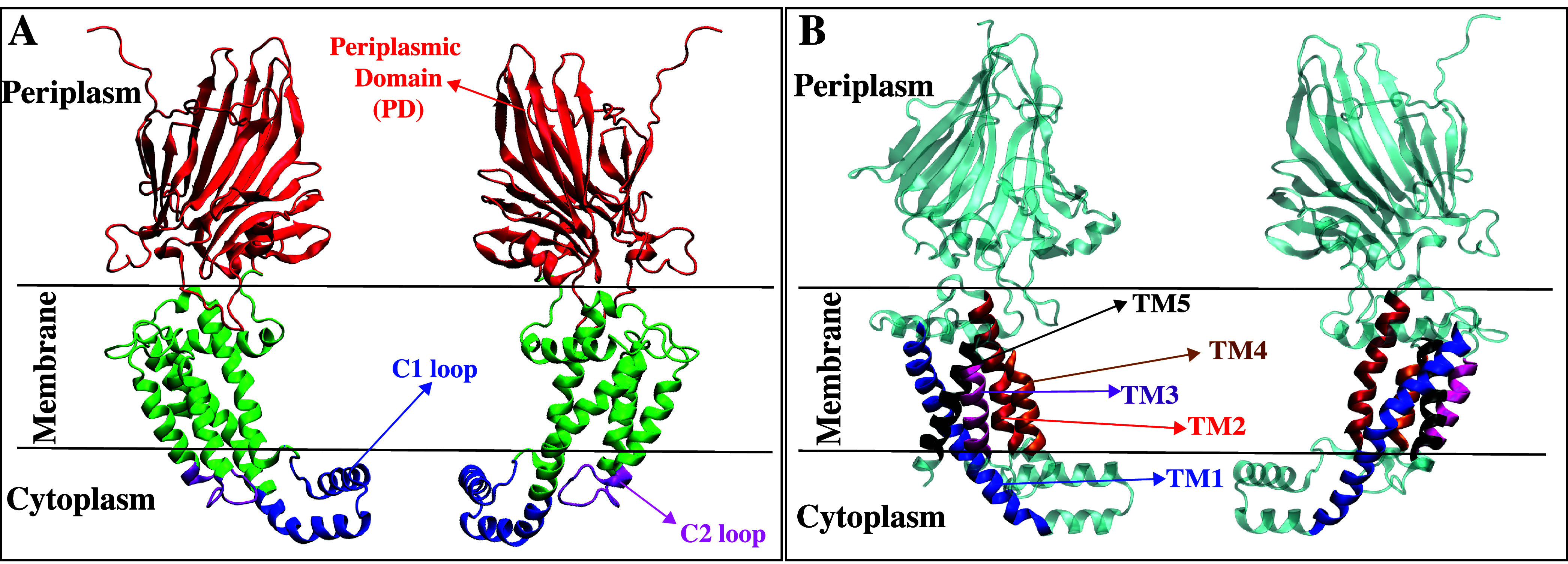
Cartoon representation of YidC (PDB: 6AL2): (A) periplasmic domain (PD), transmembrane (TM) domain, and C1 and C2 loops on the cytoplasmic side. (B) Cartoon representation of YidC’s individual TM helices: TM1 (blue), TM2 (Red), TM3 (purple), TM4 (orange), and TM5 (black).

A few experimental studies have explored the role of YidC in various microbial organisms.^36−41^ The genomes of the majority of Gram-positive microorganisms encode the YidC1 and YidC2 proteins.^39,40^ Although YidC typically exists as a dimer or tetramer under physiological conditions,^42,43^ it is discovered that YidC can also exist as a monomer in lipid bilayers.^16,44^ Recent studies suggest that the oligomeric state of YidC is influenced by its concentration. Specifically, research has demonstrated that YidC can form dimers in vivo and coexist as dimers and monomers in model lipid bilayers.^45^ The Gram-negative YidC protein possesses an additional transmembrane (TM) segment at the N-terminus and a large periplasmic domain (PD) region (Figure 1).^46,47^ Although the PD region and the additional N-terminus TM segment are not required for YidC functional activity, the PD region interacts with Sec machinery and helps to create a stable complex.^46^ The area with the C-terminal five TM segments is vital for the membrane insertase activity of Gram-negative YidC.^38^ In both Gram-negative and Gram-positive bacterial strains, the protein is firmly anchored within the lipid bilayer by interfacial aromatic residues, a cytoplasmic salt-bridge group, and a periplasmic helix enhanced with aromatic residues.^36−39^ The highly conserved arginine residue (R366) in the hydrophilic groove was found in the same locations as in Gram-positive YidC (R72), implying that the arginine residue is as important for the function of Gram-negative bacteria as it is for Gram-positive bacteria.^46^ The C-terminus of monomeric YidC interacts with the ribosomes, and the short interhelical loops C1 and C2 come into contact with the ribosomal proteins.^48^ A group of aromatic residues around R72/R366 may bind with incoming peptide during insertion into the lipid bilayer.^20,36−41^

YidC is hypothesized to facilitate membrane insertion in both Gram-negative and Gram-positive bacteria by interacting with incoming peptides through specific sites: cytoplasmic loops, hydrophobic regions, and the hydrophilic groove.^16,20,49,50^ The hydrophilic groove within the membrane core of YidC helps to increase the rate at which hydrophilic moieties of a substrate are integrated into the membrane.^51−54^

**Figure 2:**
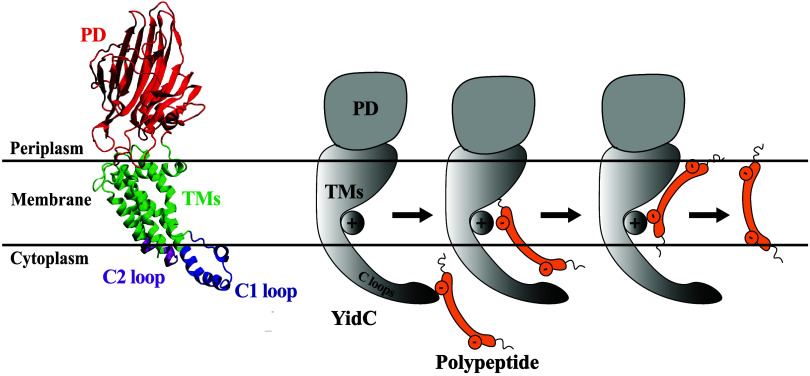
Cartoon representation of YidC and schematic illustration of the SecY-Independent insertion mechanism. The figure includes a cartoon representation of YidC, highlighting its regions: PD, TMDs, and the C1 and C2 loops. Additionally, it provides a schematic illustration of the Sec-independent insertion mechanism of a polypeptide by YidC.

During its independent insertion mechanism (Figure 2), Gram-positive YidC undergoes several conformational changes, including widening of the TM region and hydration and dehydration of the hydrophilic groove.^20^ Additionally, a broad range of interactions with the incoming protein is involved at each step of the insertion process. For instance, salt-bridge interactions with charged residues, such as R72, play a role in this process.^20^

While the YidC insertase in Gram-positive bacteria has been extensively investigated in previous studies,^16,20,49,50^ the significance of the additional PD region in Gram-negative YidC still needs to be understood. There is insufficient evidence to determine whether the PD region of YidC influences protein stability. Moreover, whether the cytoplasmic loops play analogous roles in Gram-positive and Gram-negative bacterial YidC remains unclear. Here, we investigated the structure of Gram-negative YidC using microsecond-level all-atom MD simulations. We examined the local and global conformational changes in YidC brought on by the loss of the PD region and the cytoplasmic C2 loop. While this study provides valuable data on the stability of the idle YidC monomer, it does not directly contribute to the understanding of the membrane insertion process.

## Methods

We are interested in the significance of the cytoplasmic C2 loop and extracellular PD region, as previous studies have highlighted the critical role of the C2 loop in determining YidC’s conformation and function in Gram-positive bacteria.^20,55^ We aim to learn more about the functions of the PD region (Figure 1A), which is absent in Gram-positive bacterial YidC and present in Gram-negative bacterial YidC. Accordingly, we created four systems: YidC with a PD region and C2 loop (YidC), YidC without a C2 loop (YidC ΔC2), YidC without a PD region (YidC ΔPD), and YidC without a PD region and C2 loop (YidC ΔPD ΔC2). The Gram-negative bacterial YidC crystal structure (PDB: 6al2(56)) was downloaded from the Protein Data Bank. The CHARMM36m force field,^57,58^ together with the NAMD 2.14^59^ software package was used for MD simulations run on Stampede supercomputer. Using the membrane builder on CHARMM-GUI,^60^ YidC was introduced into the lipid bilayer, solvated, and ionized. In these MD investigations, YidC was embedded in a lipid bilayer of 1-palmitoyl-2-oleoyl-*sn*-glycero-3-phosphoethanolamine (POPE) lipids. A 90 Å × 90 Å membrane layer surface was constructed along the XY plane. The protein–lipid assembly was solvated in TIP3 water^61^ with 18 Å thick layers of water on top and bottom. To neutralize the system, 0.15 M Na^+^ and Cl^–^ ions were added to the solution, with a slight modification in the number of ions. There were about ≈143,000 atoms in the final solvated system. Each system underwent energy minimization for 10,000 steps using the conjugate gradient technique^62^ before the equilibration step. Subsequently, the systems were gradually relaxed using constrained MD simulations following the standard CHARMM-GUI procedure.^60^ In the NPT ensemble at 310 K, 1 μs of equilibrium MD simulations were performed under periodic boundary conditions for each system. In the simulations, a Langevin integrator with a damping coefficient of γ = 0.5 ps^–1^ and 1 atm pressure was maintained using the Nosé–Hoover Langevin piston method.^19,63−74^ The cutoff distance for nonbonded interactions was smoothed and set between 10 and 12 Å. Long-range electrostatic interactions were computed using the Particle Mesh Ewald (PME) method.^75^

Our simulations comprised two sets for each system. For Set 1, initial simulations were run on the Stampede supercomputer for 20 ns with a time step of 2 fs. Subsequently, simulations extended on Anton 2^65^ for an additional 400 ns, with a time step of 2.5 fs. To achieve a total simulation time of 1 μs, simulations were further extended on the Stampede supercomputer for 580 ns. During Anton 2 simulations, the MTK barostat maintained a semi-isotropic pressure of 1 atm, and the Nosé–Hoover thermostat controlled a temperature of 310 K.^63,64^ Long-range electrostatic interactions were computed on Anton 2 using the fast Fourier transform (FFT) method.^76^ The conformations were collected at every 240 ps. The Anton 2 simulation trajectories were initially processed on Kollman.^65^ For Set 2, simulations were exclusively performed on the Stampede supercomputer for 1 μs each.

All trajectories were visualized and examined using the VMD software.^77^ A VMD plugin was used to analyze salt-bridge interactions by measuring the distance between the oxygen atoms of acidic residues and the nitrogen atoms of basic residues with a cutoff distance of 4 Å. Additionally, the interhelical angles were determined as the angle between the third main axes of the respective helices.^20,67,74,78,79^ The residue selection of the TM helices and other subdomains are as indicated: TM1 (355–388); TM2 (423–442); TM3 (466–479); TM4 (497–508); TM5 (511–528); C1 loop (380–420); C2 loop (480–492); and PD region (49–326) (Figure 1). We counted the number of water molecules within 5 Å of R366 to analyze the water inside the groove region. Principal component analysis (PCA) was performed for each trajectory using PRODY,^20,67,80^ considering only protein *C*_α_ atoms.

To analyze the coordinated movements of the *C*_α_ atoms, dynamic network analysis (DNA) was employed.^65,81^ This analysis calculates the correlation coefficient for the motion of each *C*_α_ atom with respect to the others using MD-TASK.^82^ Subsequently, a correlation matrix *M* was generated for each of the TM regions in all of the simulated trajectories.

To quantify the differences in correlation between a system and a reference, we calculated a difference matrix Δ using the formula:
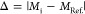
1where *M*_i_ represents the correlation matrix of interest and *M*_ref_ is the correlation matrix of a reference conformation. In this work, our point of interest was the difference between a TM region in the complete YidC structure conformation and other YidC structures. Therefore, the TM region in the wild-type YidC simulations was compared with the TM region in the other YidC simulation systems described above.

## Results and Discussion

### The Overall Protein Conformation Is Stabilized by the Presence of the C2 Loop and the PD Region

**Figure 3:**
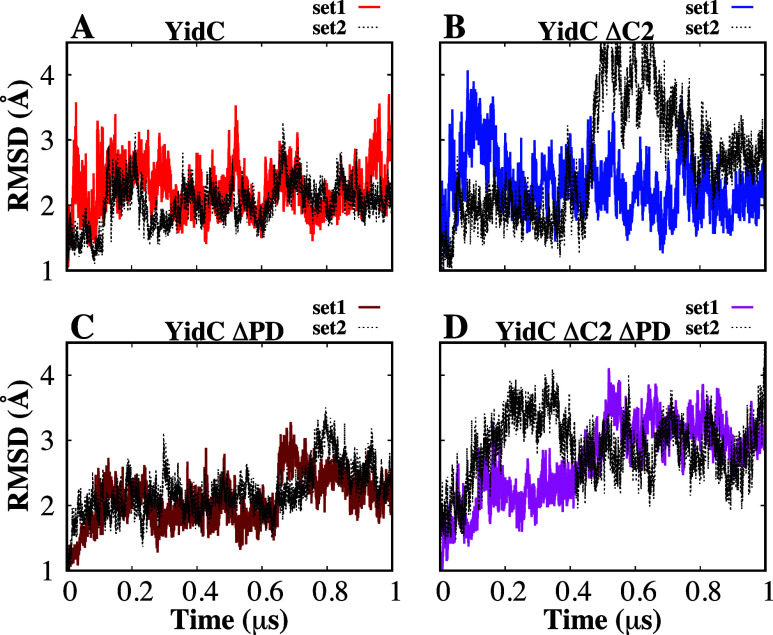
Analysis of YidC’s structural stability in the presence and absence of the PD region and C2 loop. (A–D) Root-mean-square deviation (RMSD) of YidC in different systems indicates that YidC fluctuates more in the system with the C2 loop removed compared with systems where the C2 loop is present. The simulations for each system were run twice, and the dashed lines in the graphs reflect the second run of those simulations.

The protein’s root-mean-square deviation (RMSD) was first calculated to assess its stability during equilibrium simulations. The RMSD of the YidC TM region with respect to the initial frame was computed, and the results are shown as a function of the simulation runtime (Figure 3). According to the C_α_ RMSD of the four different systems, the presence of the PD and C2 loops helps to stabilize the protein in its native state (Figure 3A). The TM RMSD of the wild-type and ΔPD systems are somewhat similar (2.1 ± 0.4 Å) in both cases when combining both sets, while this quantity is higher for ΔC2 (2.5 ± 0.8 Å) and even higher for ΔC2ΔPD (2.8 ± 0.6 Å). See also Figure S1A for the average and standard deviation of RMSD for individual trajectories and see Figure S1B for the comparison between RMSD distributions of the four systems, all of which show that the system lacking both the C2 and PD domains undergoes the largest changes, followed by the system with the PD but without the C2 domain. The system without the C2 loop shows a greater RMSD compared to the system with the C2 loop (Figure 3B and D). The combined effect of removing the PD region and the C2 loop has a higher impact on the RMSD (Figure 3D), which suggests that the PD region could be responsible for preserving the stability of the protein. However, the effect of just removing the C2 loop (Figure 3B) on protein RMSD is slightly higher than the native system (Figure S1A), but not as high as the system without the PD region and the C2 loop (Figure 3D).

This indicates that the C2 loop is more important for protein stability than the PD region, as evidenced in the system YidC ΔPD (Figure 3C). The RMSD of the system without PD is very similar to that of the wild-type YidC structure (Figure 3A). RMSF analysis for all four systems can also be found in the Supporting Information (Figure S2), similarly indicating a larger fluctuation in the transmembrane region in the absence of C2 but not PD domain. Additionally, we calculated the RMSD for the PD, TM, and C2 loop regions in the WT systems and performed hydrogen-bond analysis between the C2 loop and lipids within 5 Å. The results are shown in Figure S3.

**Figure 4:**
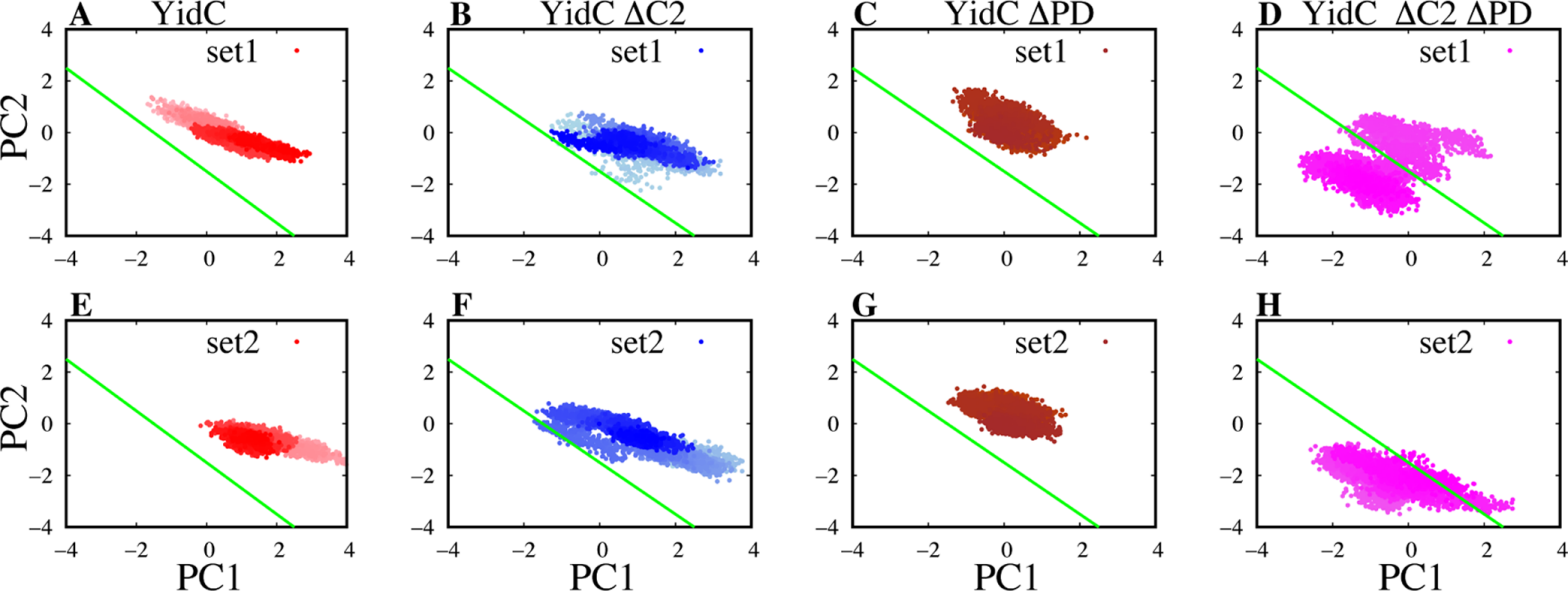
Projections of principal components (PCs) 1 and 2. (A–H) PCA findings of PC1 versus PC2 for YidC systems from set 1 simulations are shown in the top row, while the results of set 2 simulations are displayed in the bottom row. The gradation of colors in the image indicates a timeline, with lighter shades reflecting earlier points in the simulation and darker colors indicating later points. Only the PCA analysis of the TM region, present in all systems, is displayed here for consistency. To facilitate comparison, the green line on the plot indicates the difference in PC projections.

Therefore, we have concluded that the impact of eliminating the C2 loop on Gram-negative bacterial YidC is far more substantial than the effect of removing just PD regions (Figure S1). The principal component analysis (PCA) was employed to identify the most significant differences between the systems. The projections onto principal components (PCs) 1 and 2 facilitated a clear distinction between the wild-type YidC and the other systems (Figure 4).

In this particular analysis, only the YidC *C*_α_ atoms located in the TM area are considered. PC1 contributed 27.5% of the total variance, while PC2 contributed 19.1% of the total variance.

It was expected that the principal component analyses of the YidC ΔC2 and YidC ΔC2 ΔPD models would show different patterns compared to those of YidC and YidC ΔPD in PC1 and PC2 (Figure 4). This expectation is supported by the observed substantial conformational differences in the RMSD analysis (Figure S1). We also measured the (PC1, PC2) surface area sampled in these simulations from the trajectories to quantify the differential behavior of these systems. For Set 1 and 2 trajectories of the wild-type system, the area is consistently 3.2 Å^2^, while this value is 4.1 and 3.3 Å^2^ in the case of ΔPD systems (for Sets 1 and 2, respectively). This indicates a slight increase in flexibility upon the removal of the PD. The area is, however, 5.0 and 6.1 Å^2^ for Set 1 and 2 trajectories of ΔC2. Finally, for ΔC2ΔPD Set 1 and 2 trajectories, the value jumps to 8.9 and 6.1 Å^2^. Interestingly, when we combine the two sets, the area for this system jumps to 11.4 Å^2^, which is much greater than the combined value for the other systems (the largest among the other 3 systems being 7.1 Å^2^ for the ΔC2 system).

In general, the most important finding from the principal components analysis was the realization that the behavior of the YidC ΔC2 ΔPD (Figure 4 D & H) protein system was quite different from the systems with individual removal of either the PD or C2 loop, as well as the wild-type system (Figure 4).

The results of this study support the theory that the C2 loop plays a significant role in the conformational dynamics of YidC.

**Figure 5:**
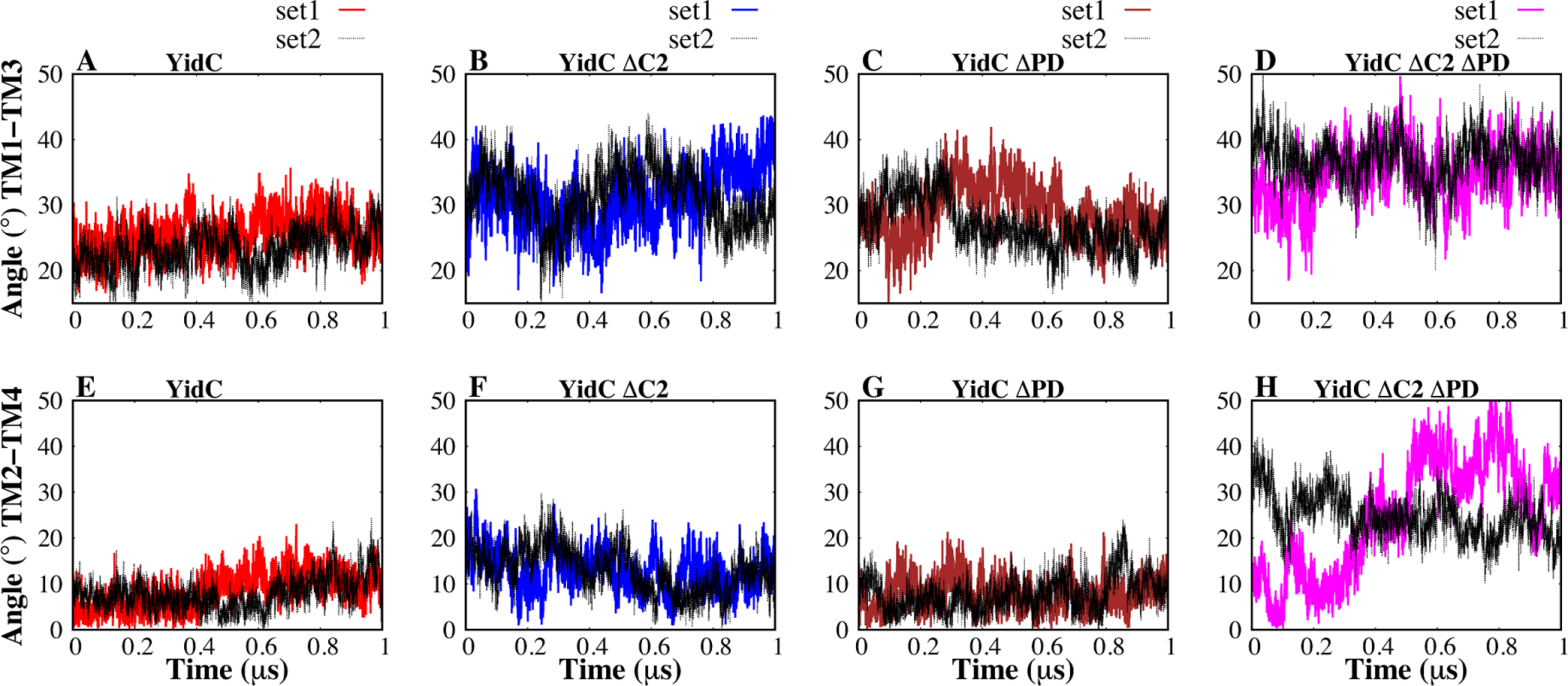
Interhelical angles between TM helices of YidC. (A–D) Interhelical angle between the protein’s TM helix 1 and helix 3. (E–H) Interhelical angle between helix 2 and helix 4 of the protein.

Previous studies have revealed that the YidC TM region is crucial for the membrane protein insertion mechanism.^20,83,84^ In order to examine the impact of deleting the PD region and C2 loop on the TM helices, the helical angle between each pair of TMs was measured in this work (Figure 5).

Compared to the wild type, we observed that the arrangement and orientation of the TM helices were modified in all other systems (Figure 5). Specifically, the helical angles between pairs of TM helices showed deviations from the wild type (Figure 5C and G), indicating changes in their spatial configuration. This alteration in the local shape of the TM helices reflects structural changes or distortions that impact the overall arrangement of the transmembrane region. The most pronounced changes were seen when both the PD and C2 loops were removed (Figure 5B, F, D, and H), suggesting the critical role of the C2 loop in maintaining the structural integrity of the TM region. We also observed a similar trend in other combinations of transmembrane helices (Figure S4).

**Figure 6:**
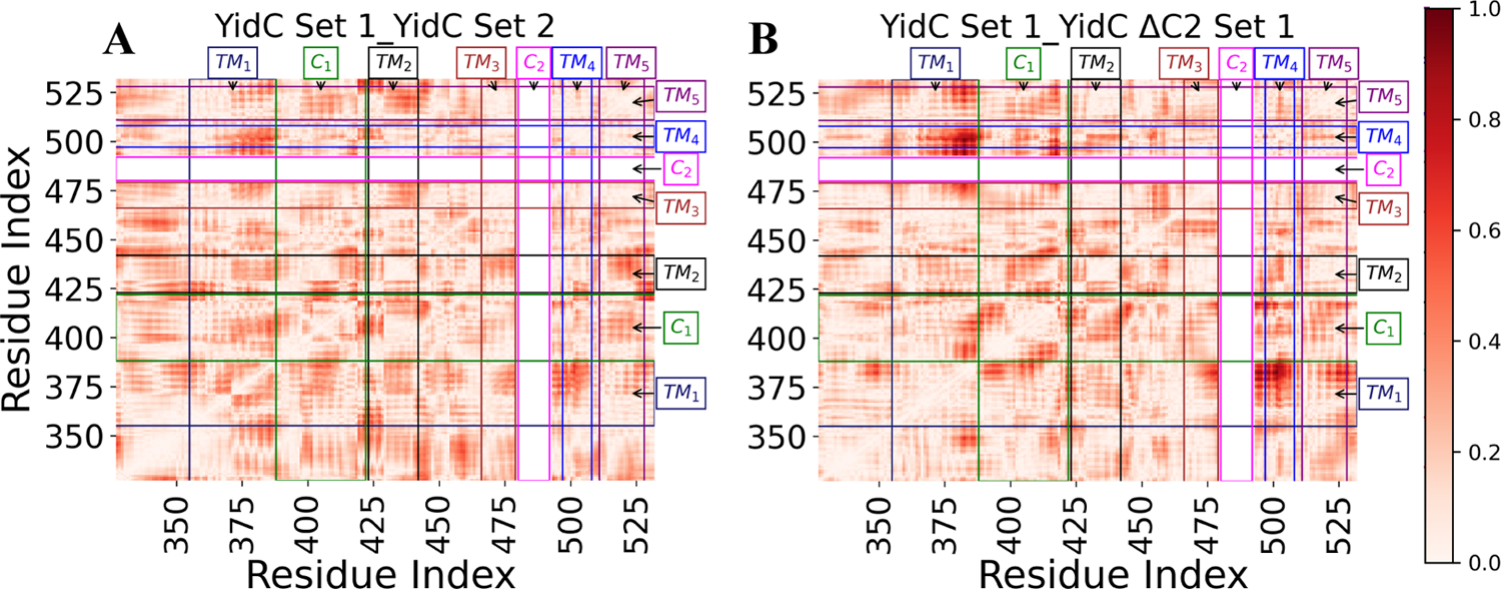
DNA analysis revealed differences in correlation between the YidC set 1 control system and the YidC ΔC2 ΔPD system examined. The theoretical maximum for the correlation difference is 2, but the observed maximum was less than 1. (A, B) Differences in correlation are shown as a red gradient, with darker red indicating a more significant difference.

Furthermore, we used dynamic network analysis (DNA), which finds the linear connections between various residue pairs, to conduct a comprehensive study of the allosteric interactions of the C2 loop and the PD region with the various protein domains. The correlation coefficient of each residue pair is shown in Figure 6. This coefficient was calculated from the trajectory with the C2 loop and PD region and then subtracted from the same quantity calculated from the trajectory without the loop and PD region systems, and the result was reported as its absolute value. The amount presented for each pair of residues measures the size of the difference in the correlation behavior of the two residues brought on by the presence of the C2 loop and the PD region in the TM region. The presence or absence of the C2 loop has been demonstrated to create significant variations in the correlations between YidC’s distinct domains. The interdomain correlations, notably between the TM/C1 loop region, vary significantly between the YidC and other systems (Figure S5).

This difference in the cross-correlation is more pronounced, especially between TM1 and TM4 (Figure 6). Based on this, we believe that the C2 loop does play a crucial role in the YidC conformational dynamics, and the absence of the C2 loop affects the conformational dynamics of the TM region. The DNA findings (Figure S5) are consistent with the early evidence for global and regional structural alterations. Overall, the results show that the C2 loop affects the behavior of the functionally essential areas of Gram-negative YidC.

### The C2 Loop and the PD Region Allosterically Influence YidC’s Other Functionally Important Regions

YidC’s U-shaped hydrophilic groove, exposed on the cytoplasmic side of the membrane bilayer, is essential for the insertion process.^51,52^ The membrane proteins enter the YidC groove through the cytoplasmic side of the membrane bilayer during the insertion process.

The groove within YidC’s transmembrane region is occupied by water molecules, which play a crucial role in the protein insertion process. As the membrane protein progresses through the insertion process, these water molecules are displaced from the groove. This displacement alters the local hydrophobicity of the groove, which may facilitate the insertion of the protein into the membrane by reducing resistance and allowing for smoother integration of the protein into the lipid bilayer.^20,36,41^

To analyze the water content of the groove inside the TM region, the number of water molecules within the groove region of the YidC protein was quantified and plotted against the simulation time.

**Figure 7:**
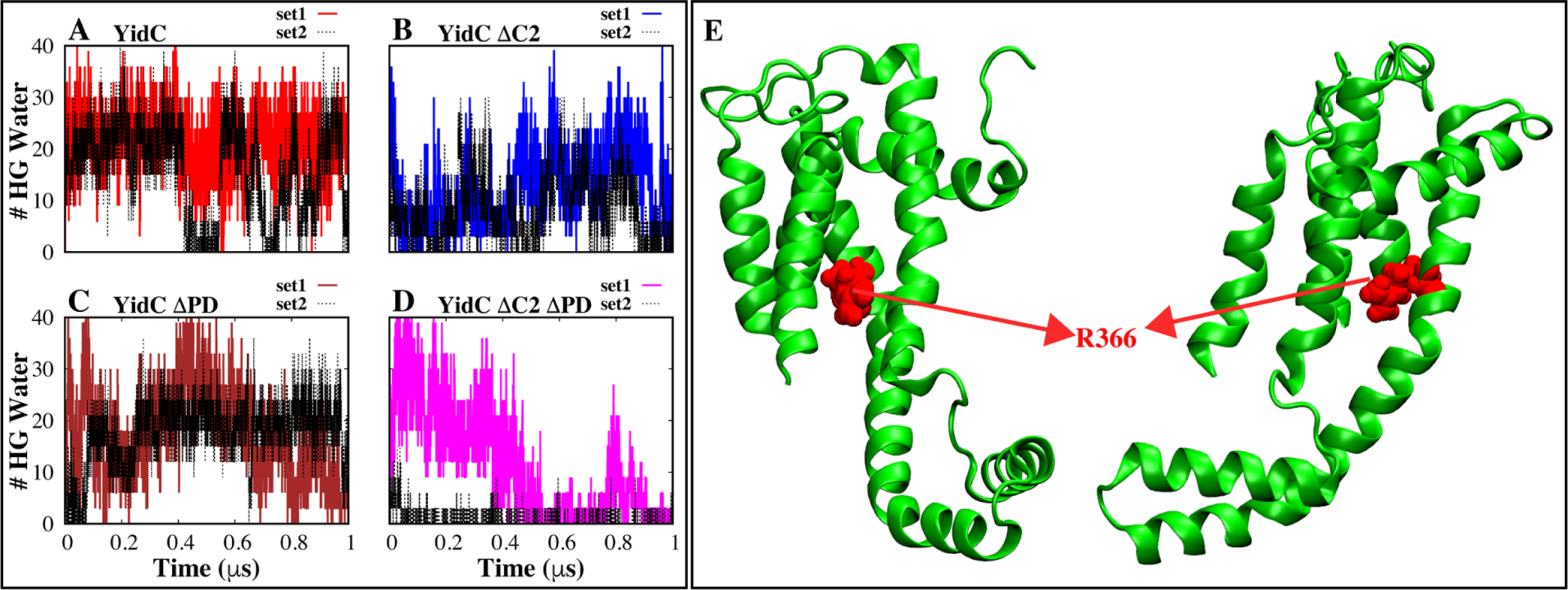
Analysis of the water inside the YidC groove. (A–D) The number of water molecules is located within 5 Å of the R366 residue inside the hydrophilic groove region of YidC in each system. (E) Graphical illustration of residue R366, which may be found in the central part of the hydrophilic groove of YidC.

The lack of the C2 loop significantly impacted the amount of water inside the groove area (Figure 7B and D). Hydrophilic contacts within the groove are crucial for maintaining the membrane protein’s position during the insertion process.^20^ Without the C2 loop, these hydrophilic interactions are reduced, leading to a decrease in the amount of water and potentially affecting the stability and efficiency of the insertion process.

This provides strong evidence that the C2 loop not only contributes to the conformational dynamics of the protein but also plays a significant role in the protein’s function. The water quantity is significantly reduced in the system YidC ΔC2 ΔPD loop compared to system YidC. Our findings lead us to infer that the removal of the PD region alone has a marginal impact on the conformational dynamics of YidC. However, when this modification is coupled with the removal of the C2 loop, the effect is significantly amplified, which could ultimately affect the insertion process.

**Figure 8:**
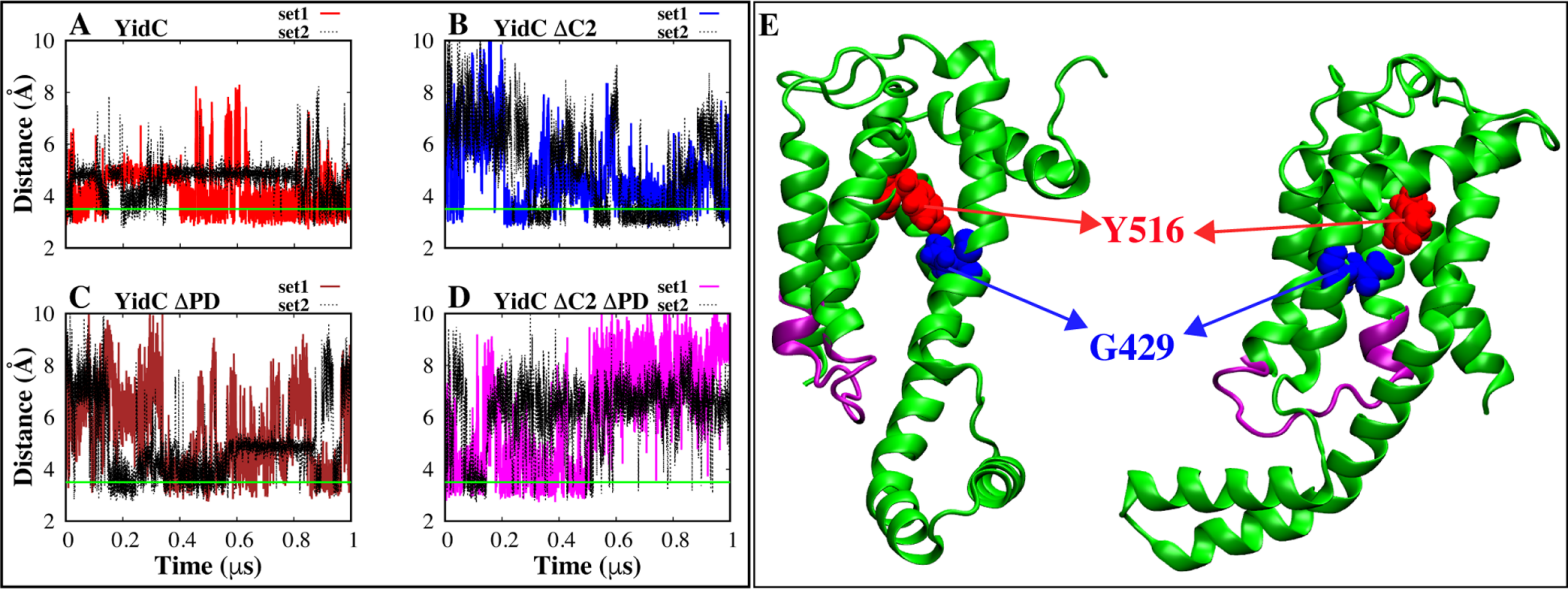
Hydrogen-bond interaction analysis between Y516 and G429, which is situated inside the YidC groove area. (A,D) Time-series hydrogen-bond distance between Y516 and G429. (E) Graphical representation of the residues that participate in the hydrogen-bond interaction.

We also found an intradomain hydrogen bond in the TM region between Y516 and G429 that is only stable in the wild-type system compared to other systems (Figure 8). Especially in the YidC ΔC2 ΔPD system, this bond is completely broken. We think this hydrogen bond is unstable in the system YidC ΔC2 ΔPD because the fluctuation of the TM region is caused by the absence of the C2 loop (Figure 8B and D). These results clearly show a link between the functionally important C2 loop and the PD region on the TM side of YidC. Furthermore, the presence of this hydrogen bond (Figure 8) appears to play a crucial role in the insertion process. As the incoming membrane protein moves along the groove and toward the periplasmic side, it breaks this link (Figure 8), causing the TM region to widen and leading the water to leave the hydrophilic groove. This results in a hydrophobic shift and increases the likelihood of membrane insertion.

To this point, we have shown that the C2 loop and PD region directly impact the structural integrity of YidC, such as its overall shape and stability. Specifically, the PD region slightly influences the conformational dynamics of the protein, which refers to the protein’s flexibility and the range of movements it can undergo. However, the absence of both the PD and the C2 loop exerts an allosteric influence on YidC’s conformational dynamics. The effect of the C2 loop deletion is substantially more significant than the effect of the PD region deletion.^46,47^

On the other hand, removing just the PD region affected the TM region’s conformational dynamics. To determine the cause of this effect, we analyzed interactions between the PD and the TM regions, which play a significant role in the stability of the protein.

### Interdomain Amino Acids Interactions Play a Key Role in the Stabilization of the TM Region

**Figure 9:**
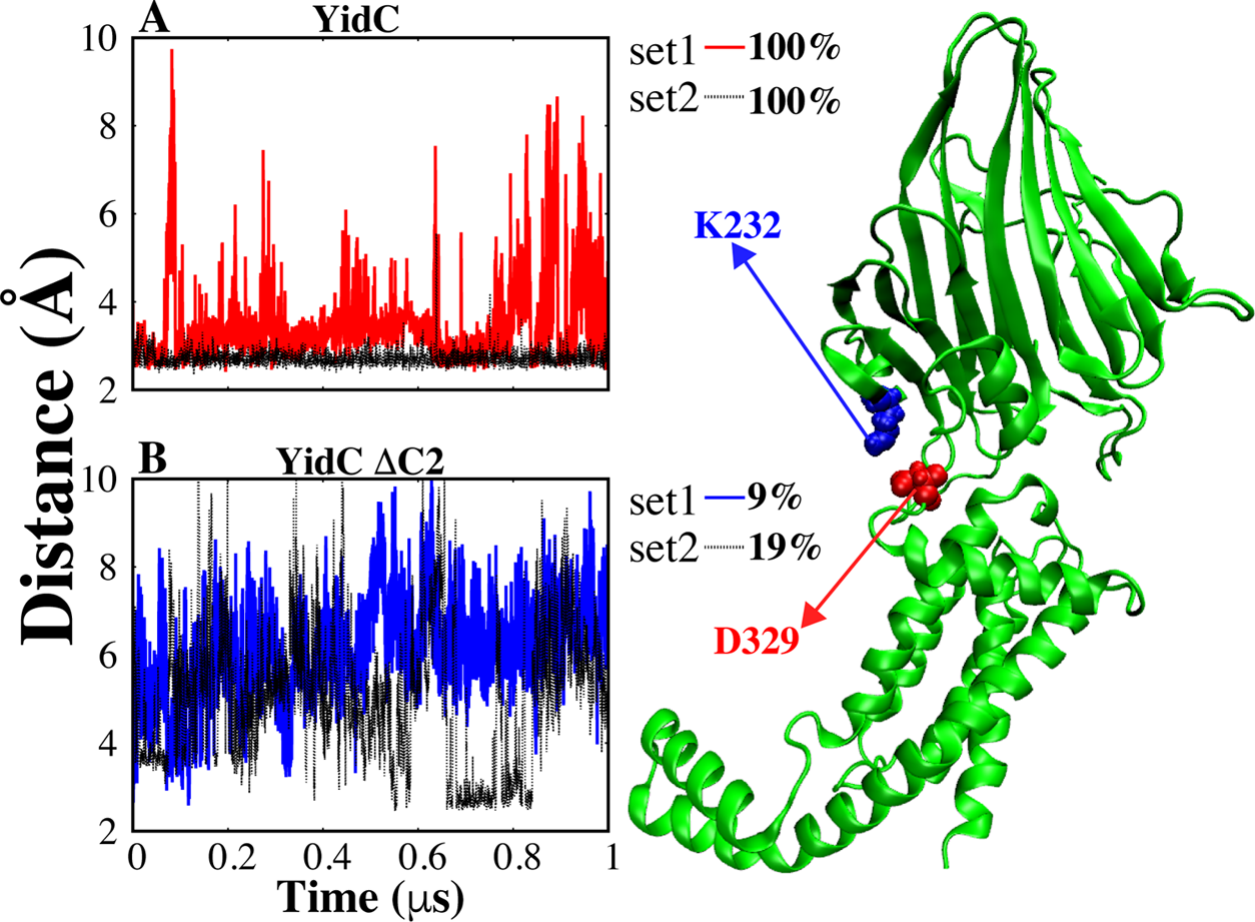
Salt–bridge formed between D329 and K232 (YidC), located between the PD and TM regions. The cartoon representation of the salt-bridge interactions that take place between the PD and TM regions (right). (A, B) Distance analysis between D329 and K232 salt-bridge, with reported occupancy of salt-bridge interaction.

Previous experimental findings led researchers to hypothesize that the interactions between the PD and TM regions of YidC are crucial for maintaining the protein’s stable state in the membrane.^46^ Through this research, we were able to identify critical hydrogen-bonding and salt-bridle interactions that take place between the PD region and the TM region. Interestingly, the hydrogen bonds formed between the PD and TM regions were only observed in the wild-type YidC system and entirely disrupted in the YidC ΔC2 system. However, there is no direct interaction between the PD and C2 loop regions, which does affect the stability of the TM region (Table. 1). We also identified a salt-bridge interaction between PD and TM regions that contributes to the stability of the TM region. However, the salt-bridge between K232 and D329 (Figure 9) is stably formed in the wild-type YidC system, this salt-bridge is disrupted in YidC ΔC2 (Figure 9B).

**Table 1:** Occupancy (%) of Interdomain H-Bonds between PD and TM Regions

	Y259-M441 (%)		A214-Y437 (%)		T327-E312 (%)	
system	set 1	set 2	set 1	set 2	set 1	set 2
YidC	40	40	66	42	81	43
YidC ΔC2	0	0	0	0	0	0
YidC ΔPD	NA	NA	NA	NA	NA	NA
YidC ΔC2 ΔPD	NA	NA	NA	NA	NA	NA

The removal of the C2 loop affects the hydrogen bond between Y516 and G429 in the TM core region, leading to increased instability in the TM region (Figure 8). This effect is supported by the observation of increased fluctuations and structural changes in the TM region of the YidC ΔC2 ΔPD mutant compared to those of the wild type. Additionally, a single salt-bridge can significantly impact the stability of the TM system due to its role in providing essential electrostatic interactions that stabilize the protein’s overall structure. The disruption of this stabilizing interaction can lead to a cascade of structural changes, affecting the stability and functionality of the entire TM region.

Based on the analysis presented above, we postulate that the hydrogen bond (Figure 8) in the protein’s groove region is essential for preserving the structural stability of the protein. The C2 loop is crucial for this hydrogen bond to remain stable in the structure. Although the YidC PD region does have a more significant influence on the protein’s structure, its contribution to the protein’s overall function is noticeably less substantial than that of the C2 loop. Even without the PD region, a normal Sec-independent insertase mechanism is possible; however, the absence of the C2 loop may have a detrimental influence on the protein’s function.

### Proposed Independent Insertion Mechanism of Gram-Negative Bacterial YidC

According to the findings and earlier hypotheses, Gram-negative bacterial YidC must likewise undergo significant conformational changes during the Sec-independent insertion procedure, much like Gram-positive bacterial YidC. During the Sec-independent insertion process, the entering membrane protein would first contact the cytoplasmic loops and then move into the hydrophilic groove, where it would join forces with R366 to create a salt-bridge. Incoming protein must be moved into YidC’s hydrophilic groove by the YidC loops on the cytoplasmic side of the bilayer. The salt-bridge between the incoming protein and R366 of YidC also contributes to the passage of the protein toward the periplasmic side, stabilizing its position within the groove. As the incoming protein moves through the groove and approaches the periplasmic side, it breaks the hydrogen bond between Y516 and G429, leading to the widening of the TM region, which results in a hydrophobic shift through dehydration of the groove.

The proton motive force (PMF) plays a critical role in facilitating the in-membrane movement of the protein. The PMF generates an electrochemical gradient across the membrane, which provides the necessary energy to drive the conformational changes in YidC and the translocation of the incoming protein. As the PMF exerts force on the protein, it promotes the movement of the protein from the hydrophilic groove into the hydrophobic core of the membrane. This movement is further assisted by hydrophobic interactions between the protein and the lipid tails of the membrane, allowing the protein to integrate smoothly into the lipid bilayer. The combined effects of the PMF and the membrane’s hydrophobic interactions ensure the efficient insertion of the protein into the membrane. Subsequently, the protein moves through the groove and enters the membrane, completing the Sec-independent insertion process.^85^

## Conclusions

This work demonstrates that both the C2 cytoplasmic loop and the PD region of YidC are crucial for the protein’s stability. Our findings indicate that the C2 loop plays a vital role in stabilizing the protein structure by influencing interactions within the transmembrane core region and between the PD and the TM region. Additionally, the presence of the C2 loop affects functional features of YidC, such as the hydration of the groove, which is essential for its Sec-independent insertion function. In contrast, the removal of the PD region shows a less dramatic effect on the overall stability but still impacts the protein’s dynamics. The combined removal of both the PD and C2 loops results in significant alterations, underscoring their synergistic importance. Further studies are needed to elucidate the precise mechanisms by which the C2 loop and PD region contribute to the Sec-independent insertion process of small single-spanning membrane proteins such as the pf3 coat protein. In the context of molecular dynamics simulations, our research highlights that both the C2 loop and the PD region are necessary for the structural stability and function of the Gram-negative YidC membrane protein, with the C2 loop having a more pronounced effect.

## Supporting Information Available

Additional analysis based on our MD simulations as discussed in the manuscript (Figures S1–S5) (Movie S1). Simulation and analysis scripts are available on GitHub Page: https://github.com/bslgroup/YidC (PDF)
