# Supplementary material for: Deciphering the Interdomain Coupling in a Gram-Negative Bacterial Membrane Insertase
Source: J Phys Chem B. 2024 Sep 27;128(40):9734–44. doi: 10.1021/acs.jpcb.4c02824 (PMC11472308; doi:10.1021/acs.jpcb.4c02824)
Supplement: Supplementary file 1 — jp4c02824_si_001.pdf [file jp4c02824_si_001.pdf]

Supporting Information: Deciphering the Inter-domain Coupling in a Gram-negative Bacterial Membrane Insertase

Adithya Polasa, Shadi A Badiee, and Mahmoud Moradi*

Department of Chemistry and Biochemistry, University of Arkansas, Fayetteville, Arkansas 72701, U.S.A.

E-mail: moradi@uark.edu

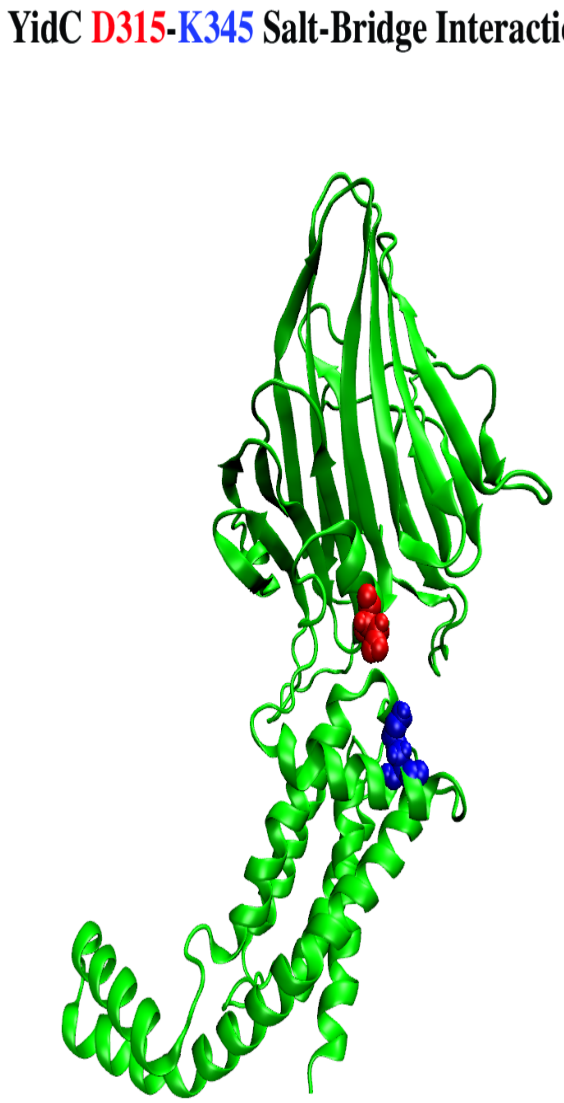

Mov. S1. Salt–bridge interaction between D315 and K345 (YidC), which is located between the PD and TM regions.

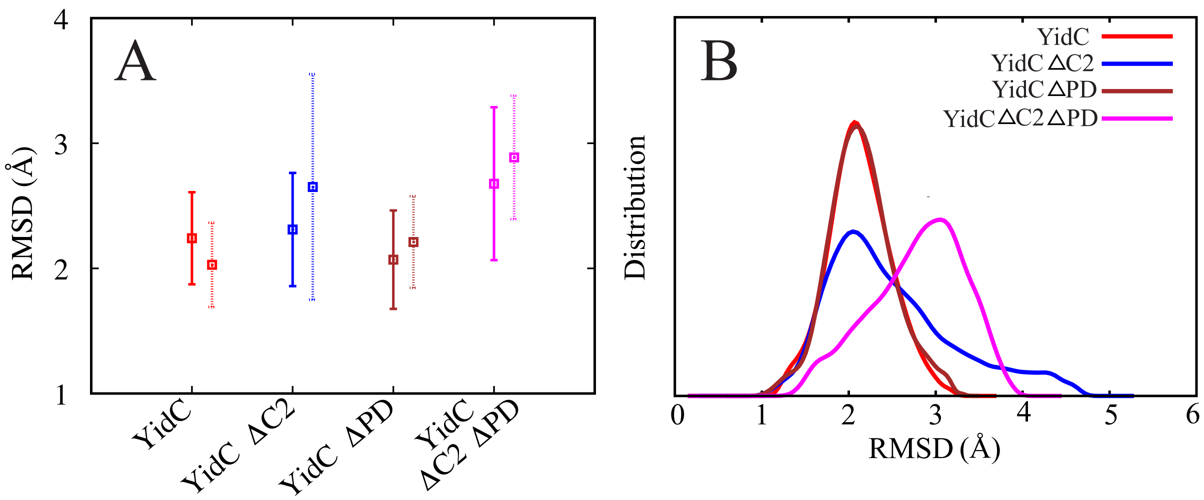

Fig. S1. Analysis of the structural stability of YidC with and without the PD region and C2 loop. (A) The average and standard deviation of the YidC's root mean square deviation (RMSD) in various systems are shown in this figure. Based on RMSD data, we have shown that YidC is more fluctuating in the system without the C2 loop and PD region than in the system with the C2 loop and PD region. The dashed lines in the graphs represent the second simulation run for each individual system. (B) RMSD distribution comparing the structural stability of YidC with and without the PD region and C2 loop. The figure shows the RMSD distribution of YidC across different systems.

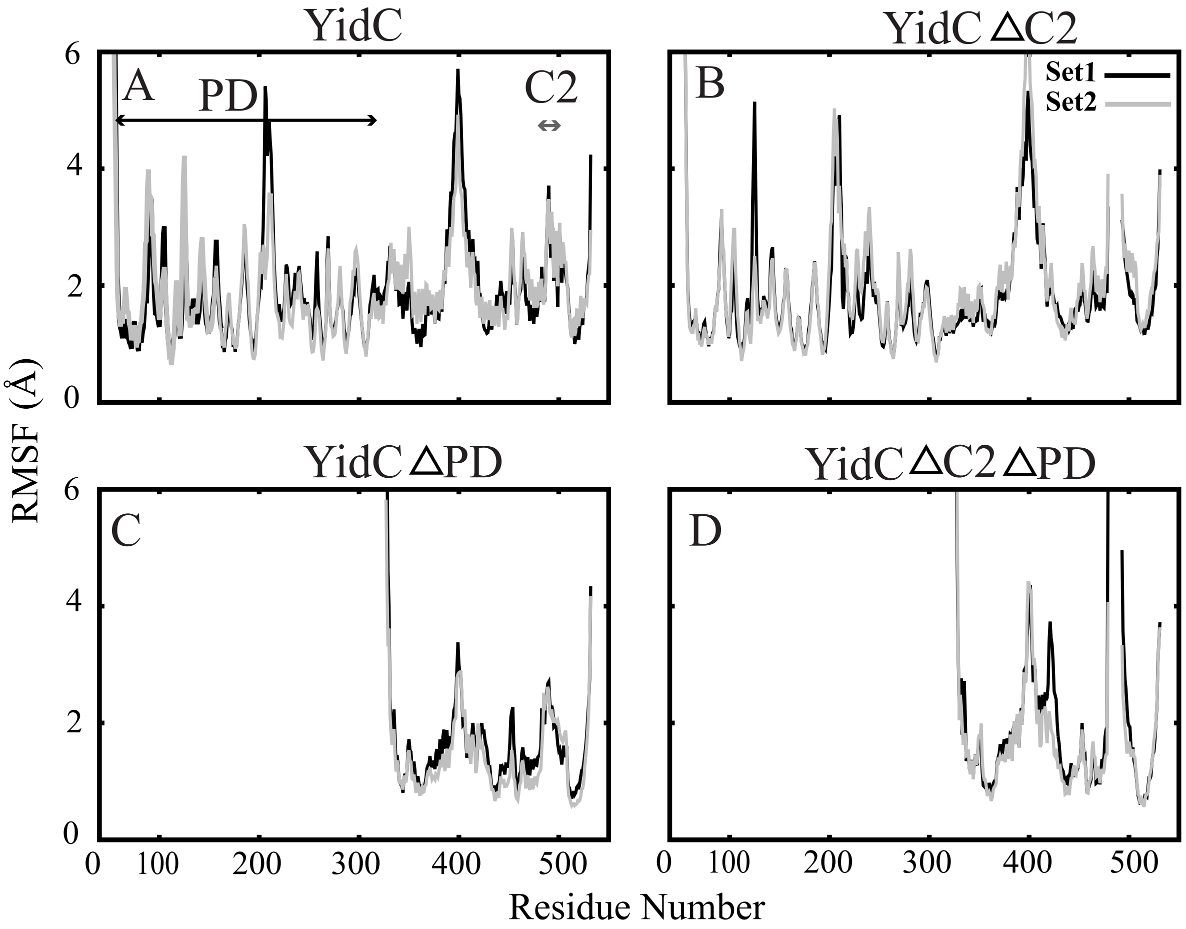

Fig. S2. RMSF calculation for all systems.

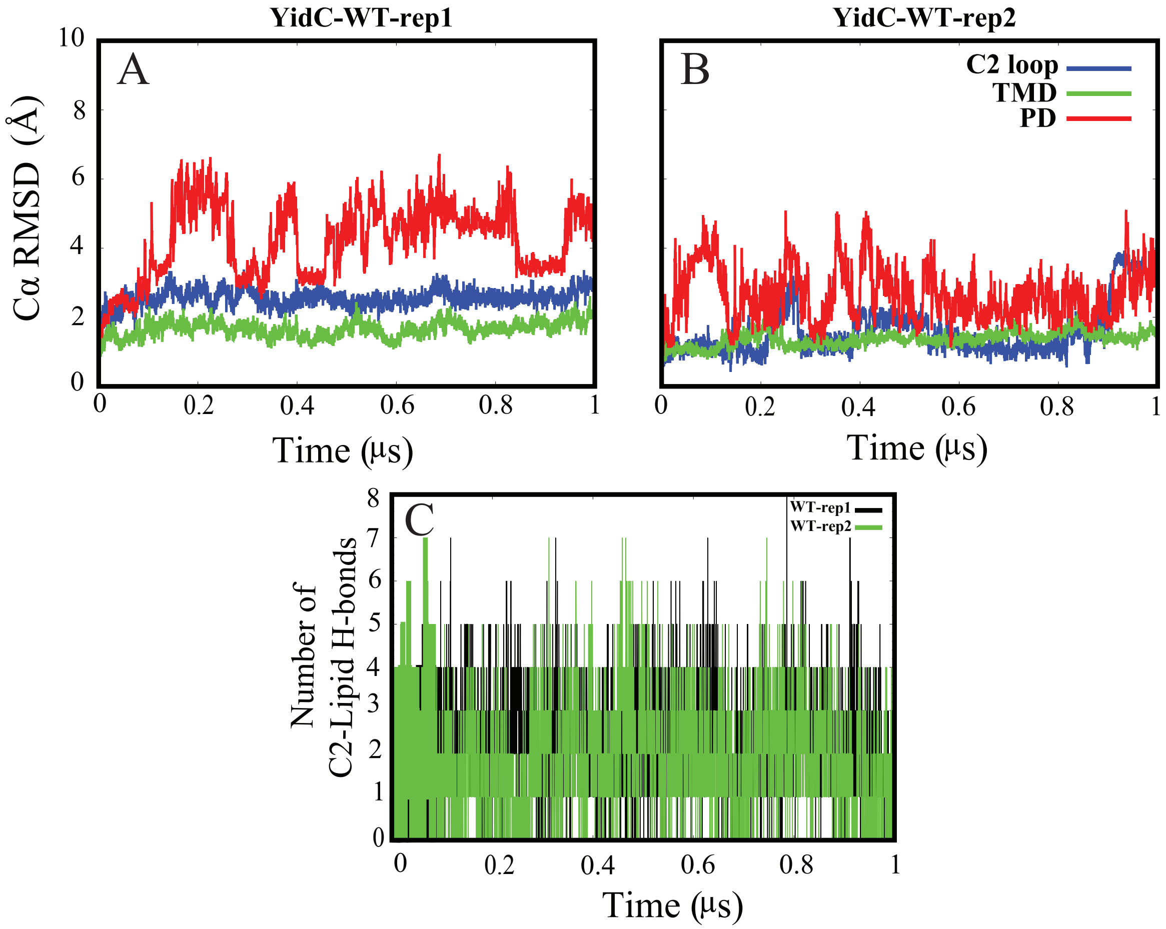

Fig. S3. RMSD calculations for the PD, TM, and C2 loop regions in the WT systems, along with hydrogen bond analysis between the C2 loop and lipids within 5 Å.

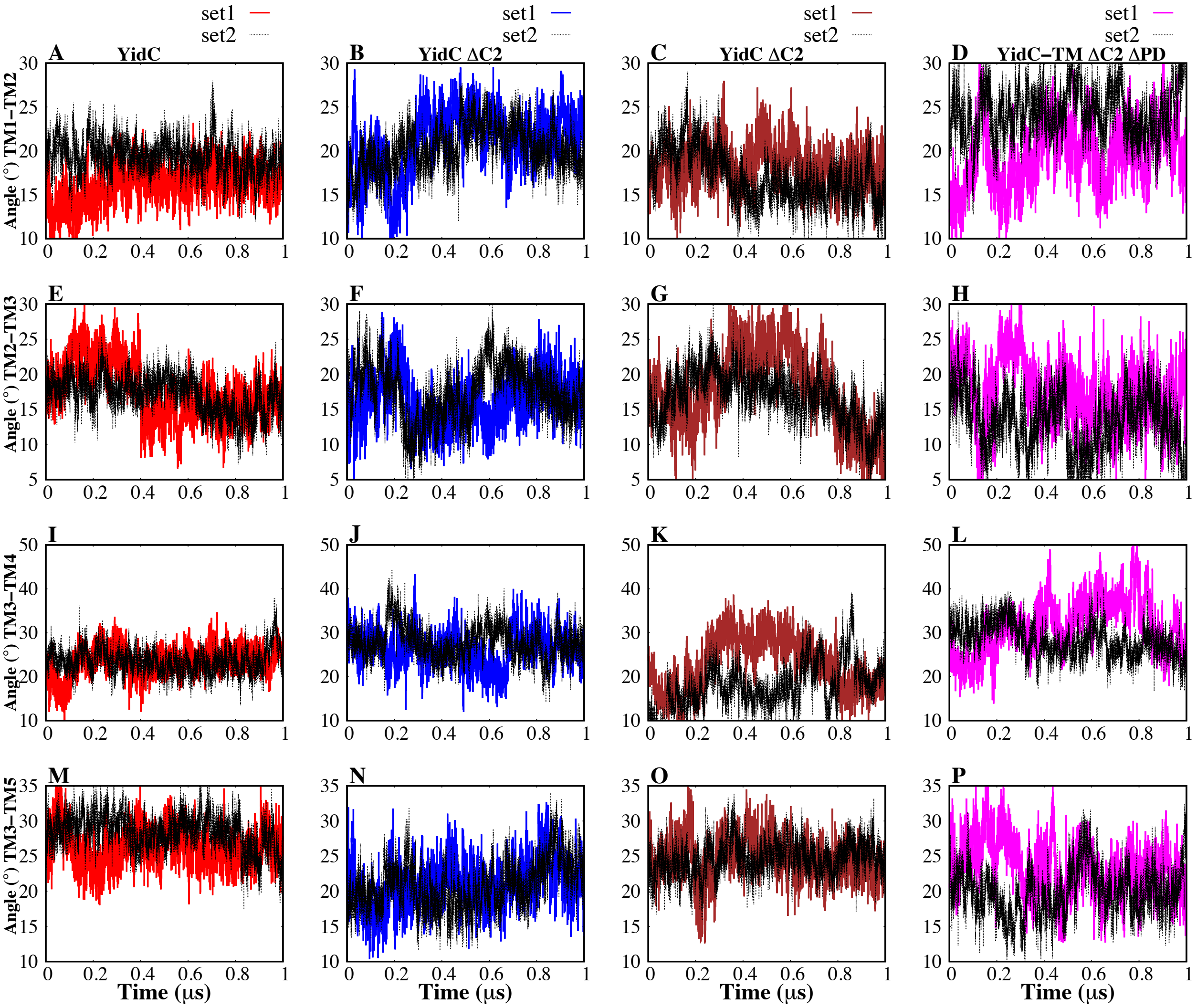

Fig. S4. Inter-helical angles between TM helices of YidC. (A–D) The inter-helical angle between the TM helix 1 and helix 2 of the protein. (E–H) The inter-helical angle between helix 2 region and helix 3 of the protein. (I–L) The inter-helical angle between the TM helix 3 and helix 4 of the protein. (M–P) The inter-helical angle between helix 3 region and helix 5 of the protein.

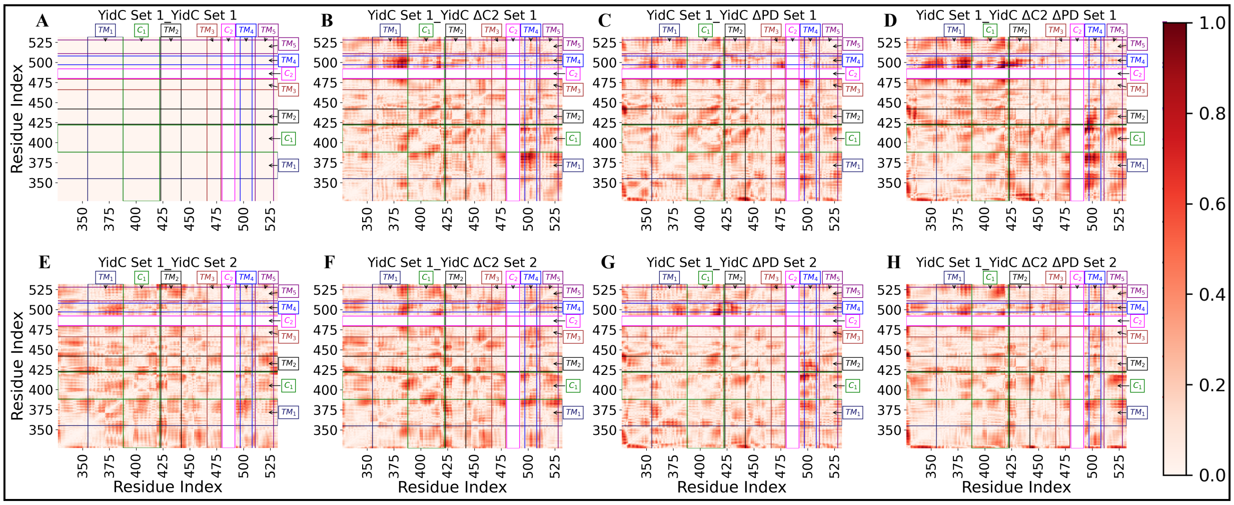

Fig. S5. The DNA analysis showed that the YidC set 1 control system and the other YidC systems that were investigated for this research have distinct disparities in their correlations. Although the highest difference in correlation that may be detected in practice was less than one, the theoretical maximum is two. (A-H) Differences in correlation are shown as a gradient of red, with deeper red representing bigger differences.
